# Knowledge, Attitude and Practices Regarding Benign Testicular Disorders in the Educated Young Men of Pakistan

**DOI:** 10.7759/cureus.1563

**Published:** 2017-08-13

**Authors:** Dua Saleem, Samra Muneer, Rajaa Fatima Younus Khan, Rohan Kumar Ochani, Syed Saadan Ahmed, Maha Begg, Tariq J Siddiqi, Syed Raza Abbas, Muhammad W Naseeb, Muhammad Osama Farooqui, Fahad H Shaikh, Ramiz Kirmani, Hurmat Ullah, Kaneez Fatima

**Affiliations:** 1 Dow Medical College, Dow University of Health Sciences (DUHS), Karachi, Pakistan; 2 DIMC; 3 Department of Internal Medicine, Dow University of Health Sciences (DUHS), Karachi, Pakistan

**Keywords:** benign testicular disorders, testicular self examination, karachi

## Abstract

Background

It has been seen that despite the increasing incidence of benign testicular disorders (BTDs), little work has been done towards its awareness among the male populace. Also, the trend of not seeking help in this regard is concerning. In this study, we aim to better perceive the level of understanding and common practices regarding BTDs among educated young men.

Methods

A cross-sectional study was carried out among two groups of ages 14-20 and 21-28 years. The inclusion criterion was that of educated males in an urban setting. Data were collected through a standardized questionnaire using cluster sampling by independent interviewers. The questionnaire consisted of four parts dealing with demographics, knowledge, attitudes and practices. Chi-square and Mann-Whitney U test were used as the primary statistical tests.

Results

The sample population consisted of an equal number of participants between the ages of 14 and 20, and between 21 and 28 years (n = 200, 50%). About half the participants (n = 215, 53.8%) were not familiar with the term BTDs. The majority (n = 324, 78.8%) of participants were not aware of symptoms of BTDs. Three-fourth of the participants believed that the subject is considered taboo in Pakistan (n = 307, 73.6%) while a majority of participants (n = 340, 85%) believed media coverage can help spread awareness of BTDs. A huge number (n = 268, 67%) thought that BTDs can cause fertility problems while one-third of them would not perform testicular self-examination (TSE) in case of pain or swelling in the scrotal region (n = 119, 29.8). The level of education and age were significantly associated with the knowledge regarding symptoms and types of BTDs.

Conclusion

Knowledge of BTDs and practices of TSE in the young educated men of Karachi are alarmingly poor. Therefore, there is an urgent need to create awareness at all levels using different strategies and platforms.

## Introduction

Diseases that affect the testes can span from being non-malignant and painless to life threatening and agonizing. Benign testicular disorders (BTDs) are the most common non-cancerous testicle problems that present as painless lumps or swellings in the scrotum [1]. However, delays in seeking help can drastically increase the chances of complications such as ischemia, necrosis, sepsis and even infertility [2]. Furthermore, in a recent study, Haas, et al. found that about 31% of all testicular cancer cases, which are classified as the most common malignancy between the ages 18 and 40 years, initially presented as a benign disorder. The study further concluded that if benign disorder of the testicles were diagnosed earlier and managed correctly, radical orchiectomy could have been prevented in majority of the cases [3].

We hypothesize that it is the lack of awareness among males which prevents them from getting an early diagnosis and treatment of BTDs. Previously, it has been shown that greater the awareness, the more likely people are to seek help [4]. Due to lack of awareness, about 39% of the people choose to delay seeking help even with obvious symptoms [5]. While there has been research done on the clinical diagnosis and management of BTDs [4, 6-7], not much work has been done regarding its awareness especially in developing countries such as Pakistan.

The aim of this research was to analyze the mindset of the educated Pakistani male population regarding BTDs, the extent of their knowledge on the subject and their practices towards dealing with such situation may it arise. The secondary objective was to establish an idea about the approach of the populace to further educate people on the various disorders of the testicles so that with early recognition and treatment, further aggravation can be avoided.

## Materials and methods

This cross-sectional study was carried out in Karachi, Pakistan during February 2017 after approval from the Institutional Review Board of Dow University of Health Sciences. The sample population consisted of young males between ages of 14 and 28 years. The study was conducted in areas of high socio-economic standards within the city and only men belonging to an educated background were included. Two separate groups were made based on age. The first group consisted of men between the ages of 14 and 20 years while the other consisted of men between 21 and 28 years. Men who were uneducated, not between 14 and 28 years old or lived in areas of low socio-economic standards were excluded from the study. The computed sample size was 384, however, 400 samples were taken equally divided between the two groups for convenient statistical comparison.

A structured standardized questionnaire was used to collect the data. The questionnaire, which comprised of four sections, contained 29 questions. Section one was to establish demographical details while the second section was to assess knowledge. Knowledge was assessed considering the familiarity with BTDs, ability to tell the difference malignant and BTDs, ability to judge their own condition based on symptoms (lumps, uneven testicle size) and to differentiate between the different types of BTDs. The third section determined the subjects’ attitudes which were gauged by: the ability to admit that BTDs are very serious, the ability to feel whether there is enough attention given to BTDs in Pakistan, the ability to admit that testicular health is a taboo subject in Pakistan and the awareness that BTDs can cause fertility problems. The fourth part of the questionnaire dealt with questions evaluating the subjects’ practices with regards to BTDs. The practice was assessed considering these elements within the survey: awareness of any injuries or symptoms in the groin, willingness to take action accordingly and willingness to spread awareness about BTDs amongst friends.

The interviewers used standard protocol with all subjects; they were given time to fill the questionnaire privately to ensure accurate results by avoiding interviewer bias. Written consent was received from all the participants before the questionnaire was filled. The questionnaires that were incomplete were discarded and no imputation methods were used to maintain an accurate representation of the views of the sample population.

Data were entered and analyzed using IBM SPSS Statistics v. 21.0 (Armonk, NY). Descriptive Statistics was used to report frequencies and proportions for the categorical responses. Chi-square test was used to check for the disparity between the categorical responses of the males belonging to two different age groups. In the case of ordinal data, the Mann-Whitney U test was used. p-values less than 0.05 were considered significant in all cases.

## Results

The sample population consisted of equal number of participants between the ages of 14 and 20 years, and between 21 and 28 years (n = 200, 50%). Table 1 shows trends regarding knowledge of BTDs, some of which are presented below. About half the participants (n = 215, 53.8%) were not familiar with the term BTDs. Similar proportions of subjects knew the difference between malignant and benign testicular disorders (n = 209, 52.3%). The majority of participants were not aware of symptoms of these disorders (n = 324, 78.8%). Epididymitis (n = 102, 25.5%) and torsion (n = 98, 24.5%) were the most recognized forms of BTDs.

**Table 1:** Knowledge regarding benign testicular disorders amongst young males in two age groups.

		Age (years)		p-value
		14-20	21-30	
How familiar are you with the term Benign Testicular Disorder?	Very little	115 (57.5%)	100 (50%)	0.15
	Moderately	69 (34.5%)	71 (35.5%)	
	Very well	16 (8%)	29 (14.5%)	
Do you know the difference between Malignant and Benign Testicular Disorders	Yes	98 (49%)	111 (55.5%)	0.254
	No	102 (51%)	89 (44.5%)	
Do you think that unequal sized testicles are associated with a higher risk for BTDs?	Yes	78 (39%)	84 (42%)	0.382
	No	122 (61%)	116 (58%)	
Do you know any symptoms of BTDs?	Yes	33 (16.5%)	57 (28.5%)	0.002
	No	167 (83.5%)	143 (71.5%)	
At what age do you believe BTDs are more common?	0-18	28 (14%)	33 (16.5%)	0.14
	18-36	99 (49.5%)	100 (50%)	
	36-54	38 (19%)	46 (23%)	
	54+	35 (17.5%)	21 (10.5%)	
Which type of BTD(s) are you aware of?				
Epididymitis	Yes	33 (16.5%)	69 (34.5%)	<0.001
	No	167 (83.5%)	131 (65.5%)	
Hydrocele	Yes	21 (10.5%)	60 (30%)	<0.001
	No	179 (89.5%)	140 (70%)	
Varicocele	Yes	17 (8.5%)	51 (25.5%)	<0.001
	No	183 (91.5%)	149 (74.5%)	
Torsion	Yes	29 (14.5%)	69 (34.5%)	<0.001
	No	171 (85.5%)	131 (65.5%)	
Other	Yes	10 (5%)	10 (5%)	0.974
	No	190 (95%)	190 (95%)	

Table 2 shows that about three-quarters of the participants believed that the subject is considered taboo in Pakistan (n = 307, 73.6%), many participants believe it is not well covered by media and public awareness (n = 296, 70.6%), and an appreciable number of participants would be open to raising awareness among friends (n = 308, 77%). The overwhelming majority (n = 340, 85%) of participants believe media coverage can help spread awareness of BTDs.

**Table 2:** Attitudes regarding benign testicular disorders amongst young males in two age groups.

		Age (years)		p-value
		14-20	21-30	
Do you think homeopathic medicine can cure a testicular disorder?	Yes	61 (30.5%)	78 (39%)	0.165
	No	139 (69.5%)	122 (61%)	
Do you think modern technologies such as cell phones and laptops have increased the incidence of testicular disorders?	Yes	95 (47.5%)	89 (44.5%)	0.682
	No	40 (20%)	49 (24.5%)	
	I don’t know	65 (32.5%)	62 (31%)	
Do you think that drugs have a role in causing BTDs?	Yes	78 (39%)	75 (37.5%)	0.362
	No	38 (19%)	39 (19.5%)	
	Maybe	64 (32%)	86 (43%)	
Do you think STDs/UTIs have a role in causing BTDs?	Yes	85 (42.5%)	88 (44%)	0.823
	No	34 (17%)	35 (17.5%)	
	Maybe	81 (40.5%)	77 (38.5%)	
Do you think BTDs can cause fertility problems?	Yes	137 (68.5%)	131 (65.5%)	0.051
	No	23 (11.5%)	20 (10%)	
	I don’t know	40 (20%)	49 (24.5%)	
Do you think testicular health is a taboo subject in Pakistan?	Yes	145 (72.5%)	151 (75.5%)	0.65
	No	55 (27.5%)	49 (24.5%)	
Do you think media can help spread awareness?	Yes	166 (83%)	174 (87%)	0.476
	No	34 (17%)	26 (13%)	
How serious do you think BTDs are?	Very little	41 (20.5%)	37 (18.5%)	0.051
	Moderately	86 (43%)	112 (56%)	
	Very well	73 (36.5%)	51 (25.5%)	
How well do you believe testicular disorders are documented in Pakistan?	Very little	154 (77%)	130 (65%)	0.052
	Moderately	36 (18%)	62 (31%)	
	Very well	10 (5%)	8 (4%)	

Table 3 shows a considerable proportion would be willing to get checked by a doctor (n = 124, 31.0%), while a similar proportion would wait for the condition to resolve itself (n = 123, 30.8%). Moreover, the majority of participants did not have any immediate relatives or acquaintances who suffered from BTDs (n = 375, 93.8%). Many (n = 260, 65.0%) would undergo surgical treatment if necessary, and a similar proportion (n = 268, 67%) think that BTDs can cause fertility problems. One-third of the participants (n = 119, 29.8%) would not perform testicular self-examination (TSE) in case of pain or swelling in the scrotal region and 6% participants had a family history of BTDs. Older participants were also significantly more likely to be willing to undergo surgical intervention (p = 0.007).

**Table 3:** Practices regarding benign testicular disorders amongst young males in two age groups.

		Age (years)		p-value
		14-20	21-30	
Have you or any of your immediate family ever suffered from BTDs?	Yes	12 (6%)	13 (6.5%)	0.949
	No	188 (94%)	187 (93.5%)	
Would you be willing to undergo surgical treatment for BTDs?	Yes	128 (64%)	132 (66%)	0.371
	No	72 (36%)	68 (34%)	
With what frequency do you get urine tested? (Tests per year)	<1	130 (65%)	131 (65.5%)	0.846
	1	50 (25%)	45 (22.5%)	
	2	16 (8%)	15 (7.5%)	
	>2	4 (2%)	9 (4.5%)	
Have you ever suffered any injury to your groin/testes?	Yes	56 (28%)	55 (27.5%)	0.816
	No	144 (72%)	145 (72.5%)	
If you were to feel pain or tenderness in your groin/testes what would your primary reaction be?	Go to the doctor	60 (30%)	64 (32%)	0.211
	Ask your parents/friends	33 (16.5%)	21 (10.5%)	
	Wait for it to resolve itself	53 (26.5%)	70 (35%)	
	Look up on the internet	49 (24.5%)	36 (18%)	
	Other	5 (2.5%)	9 (4.5%)	
If you feel any pain or swelling in your scrotum, would you self-palpate and examine it?	Yes	135 (67.5%)	146 (73%)	0.162
	No	65 (32.5%)	54 (27%)	
Would you be willing to raise awareness about BTDs among friends?	Yes	151 (75.5%)	157 (78.5%)	0.654
	No	49 (24.5%)	43 (21.5%)	

The level of education was positively associated with many fields, including but not restricted to the knowledge of the symptoms (0.023) and types (p = 0.019) of BTDs as well as awareness of their propensity to cause fertility problems in those who suffer from them (p = 0.001). Those participants that weighed more than their peers were more likely to have either suffered from BTDs or have any immediate family members or acquaintances that have.

Age was also positively associated with knowledge about types (p < 0.001) and symptoms (p = 0.002) of BTDs.

## Discussion

Our study found that more than half of the participants had little knowledge about BTDs. This finding is consistent with a previous study conducted by Roy and Casson which stated that men between the ages of 18 and 45 lacked awareness and basic knowledge about testicular disorders; its signs/symptoms and risk factors [8]. This is crucial because the incidence of BTDs is increasing around the world [9-10], yet several studies worldwide have shown that the knowledge of young men regarding this issue, whether benign or malignant, is alarmingly poor^ ^[11-13]. Moreover, it was discovered that a considerable number of participants were unwilling to perform TSE even if they felt any pain or swelling in their scrotum. These findings are worrisome because the absence of basic knowledge and unwillingness to self-examine negatively influences peoples’ preventive measures and can lead to life-threatening outcomes because of the fatal nature of testicular disorders.

Perhaps, the reason most men are in the dark regarding awareness of BTDs and TSE in Karachi is that the majority of the participants considered this topic to be a taboo. Both social and religious restrictions are placed in the Pakistani society from discussing such sensitive topics. Other developing countries such as Nigeria and Sudan also showed similar trends. Countries with low human development index are prone to display such lack of social awareness and development. Thus, the level of knowledge and awareness remains excessively inadequate in such areas of the world [14]. Current circumstances can possibly be improved by raising awareness at multiple platforms such as social media or by conducting different seminars/workshops where medical professionals can discuss the importance of TSE and consequences of late detection of BTDs. This is explained by multiple previous studies which stated, that the reasons for not performing TSE are the lack of information and guidance about the procedure and the importance of TSE, fear of embarrassment and future consequences, feeling of guilt and perceiving it as a sin [15]. This problem has to be addressed because awareness about the importance of TSE and seeking medical help for any testicular abnormality is imperative [16]. TSE is an essential factor in diagnosing BTDs at an early stage, which would help to treat them considerably [17], but unfortunately, a small proportion of men understand the importance and performance of TSE properly [18]. We found that 6% of our participants had a family history of BTDs. It is probable that they are generally more aware about the signs and symptoms of this disease. Their greater level of understanding about BTDs can potentially contribute to early diagnosis in this particular sub-group.

Moreover, we found that the respondents in our study believed that social and print media can play an instrumental role in endorsing and eradicating the taboos around BTDs which is in accordance with Xu, et al. [19]. This finding is extremely important because lack of prior knowledge about symptoms and risk factors involving BTDs was admitted by 312 respondents, and this may result in delayed medical attention which lowers the chance to salvage the affected testicles, particularly in cases involving testicular torsion [6], and to create awareness, social media can provide unlimited confidential access to an unparalleled level of information regarding diverse subjects. It is also extremely useful since the average time spent on social media by the younger generation is, on average, 7.5 hours per day [20]. If this issue is addressed by the society, it could lead to early diagnosis, significantly decreasing the mortality risk by preventing the extension of the tumor stage, particularly with the non-seminomas and more aggressive and rapidly growing testicular tumors [21].

Our results also illustrate that older age group participants had a higher knowledge of BTDs – its types and signs/symptoms. This can be explained by the fact that older age group participants were at a higher level of education, and this is significantly associated with increased awareness of BTDs. This finding is opposed by another study which states that precise knowledge about signs/symptoms or risk factors of testicular cancer did not differ significantly between various age groups, however, awareness of TSE did [8]. Our results show that knowledge regarding BTDs and self-exam interventions in Karachi should be tailored towards younger men who report lower levels of knowledge and awareness than older men. This is essential because men will then have adequate knowledge regarding BTDs hence leading to early detectability and treatment – the key to survival.

We also found that a majority of respondents believed that having BTDs could lead to fertility problems. The is critical because, BTDs, if not treated, can lead to testicular cancer and the treatment of testicular cancer can lead to sterility due to the removal of gonads and/or adjuvant chemotherapy [22]. It can further be supported by an example of epidermoid cysts which are extremely rare, yet the most common form of benign intra-testicular tumors. It is usually noted that after partial orchiectomy to cure epidermoid cysts, the majority of non-palpable testicular lesions are benign tumors, which may lead to infertility [23]. Furthermore, we also found that almost half of the respondents believed that modern technologies, such as cell phones and laptops, increased the incidence of testicular disorders. This is in accordance with the study done by Agarwal, et al., which concluded that usage of cell phones directly affected the testicles by decreasing the semen quality, sperm count, motility, and activity [24]. Awareness of the risk factors associated with BTDs is important as the longer one delays in seeking medical help, one has higher chances of not recovering the affected testicle effectively [16]. Our study also found that participants who weighed more than their peers were more likely to have either suffered from BTDs or have any immediate family members or acquaintances that have BTDs, which is consistent with another study that stated that a higher weight is seen with increasing incidences of BTDs and infertility [25-26]. This can be understood by the fact that several disorders such as epididymitis, are caused by mechanical inflammation of the epididymis due to increased fat deposits found in the thighs [27]. This finding is important because it suggests that awareness programs should include concerns towards obesity in association with BTDs.

Our study suggests various methods concerning the matter of spreading awareness which included visual and print media, and the need for posters, videos, and campaigns. Special emphasis should be made on furthering the works to increase public awareness regarding health screening, and young adults should be educated about the importance of genital examination and the detection of testicular disorders, both malignant and benign. TSE, being an important factor, should be counseled about and the skills to perform it effectively should be taught especially to adolescents, and have them familiarize themselves with what is normal for them; this can be done so by adding health and sexual education to the School/College curriculum so that the young men know the signs and symptoms that should prompt them to seek immediate medical attention. Laws regarding the prevention of diseases and health screening measures can be introduced with information campaigns by the mass media employing wide communication platforms such as various social networks and browsers since the internet is increasingly becoming the medium for people from all walks of life for self-exploration – sexual, or otherwise – for building relationships and bridging gaps between communication and the search for knowledge. Furthermore, research efforts should be made more to explore the barriers to the knowledge of BTDs and people’s beliefs regarding that and its incidence and management. The need to understand the educational needs of men is very important along with their preferred learning strategies which could be explored by focusing on particular groups and/or holding face-to-face interviews and interacting with the general populace concerning the issue. The need for early detection and understanding testicular disorders is impertinent as it can be the difference between recovery and an orchiectomy.

There are some limitations in this study which need to be considered. Given the intimate nature of the topic, few participants might have disagreed to share their personal experience about the same creating inevitable discrepancies. Secondly, our targeted population was from only one city, Karachi, which cannot be an accurate predictor of knowledge, attitude and practice of BTDs of the entire country [16], hence further studies should be done in order to determine the generalizability of these results. It is recommended that future studies should use a mixed-method approach to generate a more comprehensive data set. Moreover, participants with a family history of BTDs were included in our study; this could have possibly been a source of inherent bias. Lastly, we did not survey healthcare professionals and their attitudes and practices which are a significant contributor to the awareness of the general population, hence in future, a huge emphasis should be put on including health care professionals in order to have more substantial results.

## Conclusions

Knowledge of BTDs and practices of BTDs and TSE in the young educated men of Karachi are alarmingly poor. With the prevailing cultural communication gap about BTDs, their risks of becoming fatal would increase. Therefore, there is an urgent need to create awareness at all levels using different strategies and platforms that engage young men from across the city with the aim of increasing self-efficacy in self-examination while considering the impact of masculinity norms and backed by policy-level support.
